# Congenital Malformations

**Published:** 1874-09

**Authors:** M. D. Mooney

**Affiliations:** Taylor’s Creek, Ga.


### CONGENITAL MALFORMATIONS.

By M. D. MOONEY, M.D., of Taylor's Creek, Ga.

*Editor Atlanta Medical and Surgical Journal:*

A few weeks ago I examined a curious case of malformation. A child four years old, well grown, and perfect in form, of good, sound mind, etc., except the forearms and hands. They are in appearance those of the gopher. When the child stands erect, with arms dropped down, the palms of the hands are turned out, and have the outward and backward motion; forearms shorter than usual; hands shorter and wider than usual.

All that I could elicit from the mother was that while in utero-gestation with said child she saw a gopher on his back in the hot sun, and was sorry for the animal, and turned it over with her foot. I have used her words.

Now, is it possible that the sympathy of the mother caused the malformation, or was the malformation caused from a defective spermatozoon, as by the theory of Dr. J. A. Meek, of Jonesboro, Arkansas? I am inclined to accept the former after thirty-five years' practice in the medical profession.

Case 2d.—I examined a child of a negro woman, who had borne three well-developed children previous to this malformed child. The top of the cranium was the seat of the malformation. The top of the frontal, two parietal and occipital bones were wanting half their length; and in place of the bones there was a well-formed terrapin—soft, but well marked. This child lived four days; took nourishment for first three days, and died with well-marked congestion of the brain. I could elicit nothing from the mother that would lead to the discovery of any cause for the deformity. She was very ignorant.

---

Similia Similibus Curantur.—The latest homœopathic remedy for diabetes mellitus is said to be a decoction of Lathyrus Odoratus, or *sweet* pea.—*Boston Medical and Surgical Journal.*

